# Primary prevention of hepatic encephalopathy post-TIPS: A systematic review and meta-analysis

**DOI:** 10.1097/MD.0000000000035266

**Published:** 2023-09-22

**Authors:** Aileen Liang, Sukhman Brar, Majed Almaghrabi, Mohammad Qasim Khan, Karim Qumosani, Anouar Teriaky

**Affiliations:** a Schulich School of Medicine and Dentistry, Western University, London, Ontario, Canada; b College of Medicine, King Saud Bin Abdulaziz University for Health Sciences, Jeddah, Saudi Arabia; c King Abdullah International Medical Research Center, Jeddah, Saudi Arabia; d Department of Medicine, Division of Gastroenterology, Western University and London Health Sciences Centre, London, Ontario, Canada; e Multi-Organ Transplant Program, Western University and London Health Sciences Centre, London, Ontario, Canada.

**Keywords:** hepatic encephalopathy, lactulose, L-Ornithine-L-aspartate, rifaxmin, transjugular intrahepatic portasystemic shunt

## Abstract

**Background:**

Transjugular intrahepatic portosystemic shunt (TIPS) can be an effective treatment for cirrhotic patients who develop variceal bleeding and ascites. However, TIPS placement is associated with an increased risk of developing hepatic encephalopathy (HE). Recently, there have been efforts to use the typical medical therapies prophylactically in patients undergoing TIPS placement to prevent post-TIPS HE.

**Methods:**

We conducted literature searches in MEDLINE, Embase, CINAHL, Scopus, and Cochrane to examine studies that use prophylactic medical therapy for preventing post-TIPS HE. A narrative synthesis and grading of recommendations assessment assessment were done for all studies. Meta-analysis was performed for eligible studies using the Mantel-Haenszel method random-effects model. Nine hundred twenty-one articles were screened and 5 studies were included in the study after 2 levels of screening. The medications studied were rifaximin, lactulose, lactitol, L-Ornithine-L-aspartate (LOLA), albumin, and combination therapies.

**Results:**

Narrative results showed that lactulose, lactitol, LOLA and albumin prophylaxis were not associated with reduction in HE occurrence or mortality. A combination of rifaximin and lactulose was found to be associated with lower occurrence of HE, and the results were not different when LOLA was added. Meta-analysis (n = 3) showed that rifaximin treatment was not associated with changes in HE occurrences.

**Conclusion:**

In conclusion, a vast majority of medications were not found to be effective post-TIPS HE prophylaxis when used alone. A rifaximin and lactulose combination therapy may be beneficial. Overall, there is significant limitation in the current data and more studies are needed to yield more robust meta-analysis results in the future.

## 1. Introduction

Transjugular intrahepatic portosystemic shunt (TIPS) is an artificial shunt that can be placed to reroute blood flow past the liver to reduce portal hypertension in patients with liver cirrhosis.^[1]^ Indications for TIPS include variceal hemorrhage, refractory ascites, and hepatic hydrothorax. Studies have shown TIPS can increase liver transplantation-free survival rates of cirrhotic patients with refractory ascites.^[2]^

However, patients with TIPS are at increased risk of developing hepatic encephalopathy (HE).^[3,4]^ Up to 23% of post-TIPS patients develop HE.^[4]^ HE is thought to be primarily caused by a rise in ammonia levels after TIPS placement.^[3]^ This occurs due to decreased breakdown of ammonia as a result of bypassing liver parenchyma as well as increased intestinal ammonia production from upregulation of glutaminase.^[3]^ Clinically, HE carries a wide spectrum of presentations, ranging from covert HE to overt HE. Covert HE is detected via neuropsychological tests analyzing psychomotor speed/executive functions, while overt HE can result in mental changes such as lethargy, disorientation, confusion and coma.^[5]^ The West Haven criteria is a scale that differentiates HE into 4 grades ranging from inattention to coma.^[6]^

The management of post-TIPS HE is similar to that of patients without TIPS. Methods of management include nonabsorbable disaccharides, specific antibiotics, L-ornithine-L-aspartate (LOLA), and albumin. Lactulose, a nonabsorbable disaccharide, is an osmotic laxative. In the case of HE, lactulose can be used to decrease the production and absorption of ammonia into the bloodstream.^[7]^ Nonabsorbable antibiotics such as rifaximin and neomycin can also be used because they are able to modulate the gut microbiota by decreasing the nitrogenous compound-producing bacteria in the colon, hence decreasing overall ammonia production. LOLA, a stable salt, is a key substrate in both the synthesis of urea and the synthesis of glutamine. LOLA is therefore able to reduce HE by inducing nitrogen metabolism to decrease blood and CSF ammonia levels.^[7]^ Lastly, studies have also demonstrated that albumin infusion may reduce occurrence of overt HE in patients with cirrhosis, as albumin has an established role in plasma expansion and decreases risks of cirrhosis-related complications related to circulatory dysfunction.^[8]^ Currently, the European association for the study of the liver (EASL) (2022) recommends that patients with covert HE be treated with nonabsorbable disaccharides. Following an episode of overt HE, the EASL recommend patients be treated with lactulose titrated to 2 to 3 bowel movements/day as secondary prophylaxis. In this case, rifaximin can also be added as an adjunct to lactulose therapy.^[9]^

Despite the many options available for treating HE, no clear guidelines have been established if these drugs can be used as primary prophylaxis for HE in patients who have received TIPS. Currently, the American Association for the Study of Liver Diseases (2014) recommends no primary prophylaxis of HE in post-TIPS patients.^[6]^ This recommendation was based on 1 study that demonstrated that the prophylactic use of rifaximin or lactitol did not reduce the incidence of post-TIPS HE.^[10]^ However, recent studies have been conducted that show drugs such as LOLA or combination of lactulose and rifaximin may offer some benefit in reducing post-TIPS HE.^[2,11]^ The EASL (2022) recommends that in patients with cirrhosis and prior episodes of overt HE, rifaximin can be considered for prophylaxis prior to nonurgent TIPs placement. Other medication however, such as lactulose, require further studies.^[9]^

To the best of the authors knowledge, there has not been a systematic review that examines the possibility of medical prophylaxis for post-TIPS HE in light of these new studies. To bridge this gap in knowledge, this paper aims to systematically review the randomized and non-randomized studies that examine the use of 1 or more medical therapies for prophylaxis of post-TIPS HE.

## 2. Methods

This manuscript was developed in accordance with the Preferred Reporting Items for Systematic Reviews and Meta-Analysis guidelines.^[12]^ The protocol of this review is documented on the International prospective register of systematic reviews (PROSPERO) registry (ID: CRD42022300004).^[13]^

### 2.1. Eligibility criteria

Eligible studies examined patients 18 years or older who have undergone a TIPS procedure and have been given pharmacotherapies immediately before or after TIPS placement. Studies that include patients with preexisting hepatic encephalopathy or patients with preexisting neurological symptoms before TIPS placement were also included. No restrictions were placed on the comparator groups as there is currently no-treatment that is used for the prevention of hepatic encephalopathy post-TIPS. Eligible studies assessed the incidence of post-TIPS hepatic encephalopathy along with the grade of HE according to the West Haven Criteria if available, as well as secondary outcomes of patient hospitalization and mortality.^[5]^ In addition, study design was limited to randomized-controlled trials, cohort studies, or case-control studies published in peer-reviewed journals. Conference proceedings and case studies were not included. Studies were also limited to those published in English to mitigate risk of mistranslation.

### 2.2. Information sources

A research librarian was consulted in the development of the search strategies. Relevant documents were identified through literature searches conducted in databases MEDLINE, Embase, Cochrane Central Register of Controlled Trials (all via Ovid interface), CINAHL and Scopus. Keywords and medical subject headings (MeSH) were used to identify all relevant studies and no date restrictions were placed on the searches. Manual citation screening of included trials was also conducted to ensure a comprehensive search. Complete search strategy is available in (Appendix 1, Supplemental Digital Content, http://links.lww.com/MD/J921).

### 2.3. Study selection and screening

All literature search results were uploaded to Covidence software and duplicates were removed through an automated duplication check conducted by Covidence and a manual duplicate check conducted by reviewers (AL and SB).

Two reviewers (AL and SB) independently conducted a title abstract screening of the eligible retrieved articles using Covidence. Potentially relevant studies then underwent full-text screening by both reviewers independently. After each level of screening, conflicts were resolved during a consensus meeting between the 2 reviewers. Cohen kappa (K) coefficient was calculated for each level of screening to assess for inter-rater reliability in screening.

### 2.4. Data items

Data was extracted from the included studies independently by 2 reviewers using Excel. Any discrepancies were resolved during a consensus meeting between the 2 reviewers. Extracted data pertained to study characteristics (authors, publication date, study design, location, number of participants), participant demographics (age, sex, model for end-stage liver disease score, type of liver disease, indication for TIPS, previous episode of HE and treatments), intervention (type of medication, dosage, route, duration), and measured outcomes (incidence and grade of HE, hospitalization, all-cause mortality).

The main outcome was to determine whether pharmacotherapies such as rifaximin and lactulose administered immediately before or after a TIPS procedure reduced the incidence of post-TIPS hepatic encephalopathy. Secondary outcomes evaluated whether the pharmacotherapies had any impact on patient mortality and hospitalization.

### 2.5. Risk of bias assessment

For randomized studies, the second version of the Cochrane risk of-bias tool for randomized trials (RoB 2) was used to assess the risk of bias in all the studies analyzed in this paper. RoB2 will identify bias in the following domains: Risk of bias arising from the randomization process; Bias due to deviations from intended interventions; Bias due to missing outcome data; Bias in measurement of the outcome, and; Bias in selection of the reported result.^[14]^

For non-randomized studies, the risk of bias in non-randomized studies of interventions (ROBINS-I) tool was used to assess the risk of bias. ROBINS-I identified the following domains: Bias due to confounding; Bias in the selection of participants into the study; Bias in classification of interventions; Bias due to deviations from intended interventions; Bias due to missing data; Bias in measurement of outcomes, and; Bias in the selection of the reported result.^[15]^ Complete risk of-bias summary plots are in (Appendix 1, Supplemental Digital Content, http://links.lww.com/MD/J923).

### 2.6. Data synthesis methods

Two reviewers (AL and SB) sequentially provided a narrative descriptive synthesis of data based on predefined criteria using synthesis without meta-analysis guidelines.^[16]^ Certainty of evidence was then assessed using the grading of recommendations assessment, development and evaluation (GRADE) across the domains of risk of bias, inconsistency, indirectness, and imprecision.^[17]^

Meta-analysis for data that was comparable across interventions and sufficiently homogenous was conducted using RStudio version 1.4.110. The meta and metasens function package was used to develop quantitative measurements and graphics. Mantel-Haenszel random-effects risk ratio (RR) was obtained. Ninety-five percentage confidence intervals were obtained and heterogeneity between trials were assessed by a chi-squared test for heterogeneity. A *P* value of < .05 was considered statistically significant. Forest plots were generated for primary outcomes with 95% confidence intervals.

### 2.7. Ethics review

Ethical approval was not necessary as this study was a systematic review and did not involve patients.

## 3. Results

### 3.1. Study selection and characteristics

**Figure 1.**
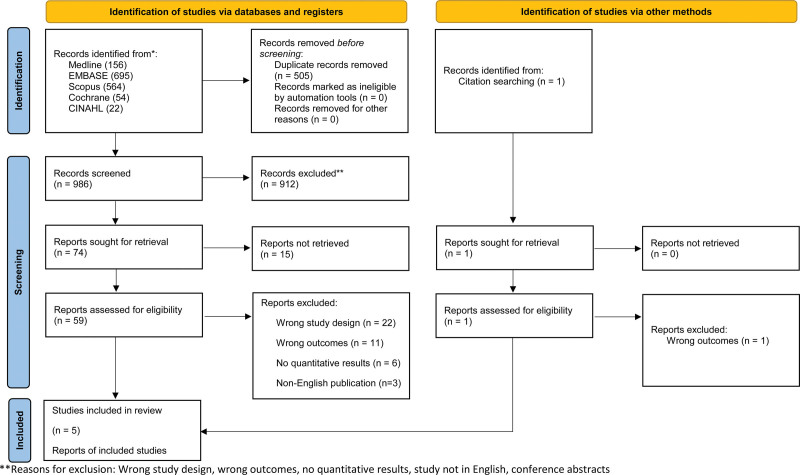
PRISMA flow diagram of screening process. **Reasons for exclusion: Wrong study design, wrong outcomes, no quantitative results, study not in English, conference abstracts.

The PRISMA flow diagram of screening process is shown in Figure 1. The search strategy yielded 986 results that were published between 1983 and 2023. After title and abstract screening, 74 studies remained and underwent full-text screening. Through full-text screening, 3 randomized-controlled trials, 1 prospective cohort study, and 1 retrospective cohort study met the inclusion criteria. The 5 included studies evaluated 1 or more medical prophylaxis therapy for prevention of hepatic encephalopathy from immediately before to within a year of the TIPS procedure.^[10,11,18–20]^ The medical therapies examined were LOLA, rifaximin, lactulose, lactitol, albumin, and various combinations. The characteristics of the included studies are shown in Table 1. GRADE tables summarize key narrative findings regarding the presence of post-TIPS HE (Table 2), grade of post-TIPS HE, and secondary outcomes of rehospitalization and mortality (Table 3).

**Table 1:** Characteristics of included studies.

First Author and Year	Study Type	Location	n	Age (mean ± SD or median (range))	Sex (male/female)	MELD Score (mean ± SD or median (range))	Type of liver disease	Indication for TIPS	Previous HE	Medication	Treatment Details
Bai 2014	RCT	China	40	Treatment: 49.7 ± 10.1 Control: 45.4 ± 9.6	Treatment: 17/4 Control: 17/2	Treatment: 12.71 ± 3.94 Control: 12.11 ± 3.11	Treatment: Viral (16), Others (5) Control: Viral (17), Others (2)	Treatment: ascites (20), bleeding (1) Control: ascites (16), bleeding (3)	Excluded	LOLA	IV 30 g daily in 500 mL of glucose 5% for 7 days
Bureau 2021	RCT	USA	197	Treatment: 61 ± 9 Control: 58 ± 8	Treatment: 73/24 Control: 79/21	Treatment: 12 ± 4 Control: 12 ± 4	Treatment: alcohol (83), others (14) Control: alcohol (87), others (13)	Treatment: ascites (82), others (15) Control: ascites (78) others (22)	Treatment: 12 Control: 13	Rifaxamin	Oral 3 200 mg capsules BID for 183 days after
Riggio 2016	Prospective cohort study	Italy	68	Treatment: 57.7 ± 10 Control: 55.2 ± 10.7	Treatment: 17/6 Control: 28/17	Treatment: 11.5 ± 3.3 Control: 10.4 ± 4.2	Treatment: alcohol (8), viral (9), others (6) Control: alcohol (18), viral (18), others (9)	Treatment: ascites (10), bleeding (13) Control: ascites (14), bleeding (21)	Treatment: 0 Control: 9	Albumin	IV 1g/Kg body weight for the first 2 days after TIPS followed by 0.5 g/Kg body weight at day 4 and then 0.5 g/Kg body weight once a week for 3 weeks
Riggio 2005	RCT	Italy	75	56.8 ± 10.8	49/26	8.9 ± 5.3	Alcohol (25), others (50)	Ascites (25), bleeding (50)	11	Lactitol, rifaximin	Lactitol oral 60 ml daily for 4 weeks, rifaximin oral 500 mg TID for 4 weeks
Seifert 2021	Retrospective cohort study	Germany	233	58 (19-80)	142/91	14 ± 7.2	NAFLD (20), alcohol (120), viral (22), others (71)	Ascites (115), bleeding (77), others(41)	49	Rifaximin, lactulose, LOLA	Rifaximin oral 500mg BID for 12 months, lactulose rectal dosed individually at 2-3 loose stools per day for 12 months, LOLA oral 3-6g TID for 12 months

HE = hepatic encephalopathy, LOLA = L-orithine-L-aspartate, MELD = model for end-stage liver disease, RCT = randomized-controlled trial, TIPS = transjugular intrahepatic portosystemic shunt.

**Table 2:** Development of Post-TIPS HE after prophylactic use of medications.

Certainty assessment						Impact	Importance
No of studies	Study design	Risk of bias	Inconsistency	Indirectness	Imprecision		
Rifaximin							
3	Randomized trials, observational studies	Not serious	Serious^a^	Not serious	Not serious	Prophylaxis with rifaximin alone likely does not reduce risk of HE in patients post-TIPS.	CRITICAL
Lactulose							
1	Observational study	Not serious	Not serious	Not serious	Not serious	Prophylaxis with lactulose alone did not reduce post-TIPS HE compared to no-treatment group. Further research is needed.	Critical
Lactitol							
1	Randomized trials	Not serious	Not serious	Not serious	Not serious	Treatment with lactitol was not effective as prophylaxis against post-TIPS HE.	Critical
Albumin							
1	Randomized trials	Not serious	Not serious	Not serious	Not serious	Prophylactic use of albumin may not be effective as prophylaxis for post-TIPS HE. More studies need to be conducted on the topic.	Critical
LOLA							
1	Randomized trials	Not serious	Not serious	Not serious	Not serious	Prophylactic use of LOLA may help prevent post-TIPS minimal HE and may reduce cases of post-TIPs severe overt HE. Further research is needed on the topic.	Critical
Rifaximin + Lactulose							
1	Observational studies	Not serious	Not serious	Not serious	Not serious	A combination of lactulose and rifaximin might be effective for prophylaxis against post-TIPS HE.	Critical
Rifaximin + Lactulose + LOLA							
1	Observational studies	Not serious	Not serious	Not serious	Not serious	Prophylaxis of post-TIPs HE with a combination of lactulose, rifaximin and LOLA may not be effective.	Critical

HE = hepatic encephalopathy, LOLA = L-orithine-L-aspartate, TIPS = transjugular intrahepatic portosystemic shunt.

a Bureau et al (2021) found that rifaximin prophylaxis was associated with a decreased risk of developing overt HE 168 days after TIPS. Riggio et al (2005), however, found that rifaximin prophylaxis was not effective in preventing HE 1 month after TIPS.

**Table 3:** Secondary outcomes including the grade of post-TIPS HE, rehospitalization and mortality post-TIPS after prophylactic use of medications.

Grade of Post-TIPS HE							
Certainty assessment						Impact	Importance
No of studies	Study design	Risk of bias	Inconsistency	Indirectness	Imprecision		
Rifaximin							
2	Randomized trials	Not serious	Not serious	Not serious	Not serious	More research is needed to clarify whether prophylaxis with rifaximin alone decreases incidence of severe grade HE.	Important
Lactitol							
1	Randomized trials	Not serious	Not serious	Not serious	Not serious	Prophylactic use of lactitol did not reduce the number of severe HE episodes (grade 3 and 4) compared to the no-treatment group.	Important
Rehospitalization post-TIPs							
1	Randomized trials	Serious^a^	Not serious	Not serious	Not serious	Prophylaxis with rifaximin alone may decrease rehospitalization after TIPS placement, however more research is needed.	Important
Mortality Post-TIPS							
2	Randomized trials	Not serious	Not serious	Not serious	Not serious	It is unclear whether rifaximin monotherapy reduces mortality in patients post-TIPS. Further research is needed.	Important
Lactitol							
1	Randomized trials	Not serious	Not serious	Not serious	Not serious	Prophylactic use of lactitol did not reduce mortality when compared to the no-treatment group.	Important
Albumin							
1	Randomized trials	Not serious	Not serious	Not serious	Not serious	Prophylactic use of albumin may not be effective as prophylaxis for reducing mortality. More studies need to be conducted on the topic.	Important
LOLA							
1	Randomized trials	Not serious	Not serious	Not serious	Not serious	Prophylactic use of LOLA does not reduce post-TIPs mortality when compared to control.	Important

HE = hepatic encephalopathy, LOLA = L-orithine-L-aspartate, TIPS = transjugular intrahepatic portosystemic shunt.

a High risk of bias due to missing outcome data.

### 3.2. Rifaximin

#### 3.2.1. Post-TIPS HE.

Seifert et al (2021) conducted a retrospective study where they found that all patients who had received rifaximin monotherapy (n = 6/233) developed post-TIPS HE. Patients were treated with rifaximin 72 hours prior to TIPS and for 12 months after TIPS. Riggio et al (2005) conducted a randomized-controlled trial (RCT) where they also found that patients treated with rifaximin for 4 weeks after TIPS and patients that received no-treatment had similar 1-month post-TIPS HE occurrence (8/25 patients in rifaximin group vs 8/25 patients in no-treatment group; *P* = .97).

However, Bureau et al (2021) also conducted an RCT where patients received rifaximin treatment 2 weeks before and 168 days after TIPS. They determined that post-TIPS HE occurrence for up to 6-month after TIPS did differ between the rifaximin and placebo group. An episode of overt HE was observed in 32 of 93 patients in the rifaximin group (34%, Cl 25%–44%) and 49 of 93 patients in the placebo group (53%, Cl 43%–63%), with an estimated risk difference of −18 percentage points (Cl −32% to −4 %, *P* = .012).

#### 3.2.2. Grade of HE.

The effects of rifaximin monotherapy on the grade of post-TIPS HE remains unclear. Bureau et al (2021) found that when compared to patients in placebo group, patients treated with rifaximin developed less grade 2 (78% vs 69%) and grade 3 HE (16% vs 25%), but these results were not shown to be statistically significant (*P* values not reported). However, Riggio et al (2005) found that the number of grade 3 and 4 post-TIPS HE episodes in the patient groups treated with rifaximin remained similar to the no-treatment group (rifaximin group = 5/25; no-treatment group = 3/25; *P* = .71).

#### 3.2.3. Rehospitalization and mortality.

Bureau et al (2021) reported incidences of gastrointestinal bleeding (1.1% vs 3.2%), acute kidney injury (24.7% vs 29.0%), hepatocellular carcinoma (2.2% vs 3.2%), liver transplantation (2.2% vs 5.4%) and all-cause mortality (11% vs 13%) in post-TIPs patients treated with rifaximin and placebo. The authors tested differences between groups for each event for significance using a x2 or Fisher exact test. They reported none of the differences were statistically significant (*P* > .05, exact value not reported). Riggio et al (2005) reported 2 patient deaths in the rifaximin group versus 1 death in the no-treatment group (*P* > .05, exact value not reported).

### 3.3. Lactulose

#### 3.3.1. Post-TIPS HE.

In the retrospective review conducted by Seifert et al (2021), lactulose monotherapy was given as HE prophylaxis 3 days before and up to 12 months after TIPS placement. They found that prophylactic lactulose monotherapy did not affect that frequency of post-TIPS HE development within 12 months after TIPS placement when compared to the no-treatment group (50.6% vs 53.7%, *P* = .808). They concluded that lactulose monotherapy did not have a prophylactic effect.

### 3.4. Lactitol

#### 3.4.1. Post-TIPS HE, grade, mortality.

The RCT conducted by Riggio et al (2005) found that treatment with lactitol was not effective as prophylaxis against post-TIPS HE. Patients were given 60 mL of lactitol per day, for up to 4 weeks after TIPS placement. The 1-month incidence of post-TIPS HE in patients was similar in the no-treatment and lactitol group (n = 8/25 vs n = 9/25, *P* = .97). The number of grade 3 and 4 HE episodes occurring in each group was also similar between the no-treatment and lactitol group (lactitol group = 5, no-treatment group = 3, *P* = .71).

In the lactitol group, 2 patients died within the first month after TIPS placement compared to the 1 patient in the no-treatment group. Statistics were not reported.

### 3.5. Albumin

#### 3.5.1. Post-TIPS HE, mortality.

Riggio et al (2016) conducted an RCT where 23 patients admitted for TIPS were prophylactically treated with 1 g/kg body weight of albumin for the first 2 days post-TIPS followed by 0.5 g/Kg body weight on day 4, day 7, and then once a week for 3 weeks. The incidence of HE 1-month to 6-month post-TIPS was compared with the incidence of HE of patients from a previous RCT (Riggio et al, 2010) following the same protocol except treated with no pharmacological treatment. In the albumin group, HE occurred in 34% (n = 8/23) patients 1-month post-TIPS, while in the control group, HE occurred in 31% (n = 14/45) patients 1-month post-TIPS (*P* > .05, exact value not reported). They concluded no differences in the occurrence of HE were observed between the 2 groups.

Riggio et al, (2016) also found no statistically significant differences in survival between the 2 groups. In the albumin group, 22% (n = 5/23) of patients died and in the control group, 18% (n = 8/45) of patients died. Specific *P* values not reported.

### 3.6. LOLA

#### 3.6.1. Post-TIPS HE.

Bai et al (2014) conducted a RCT where patients were randomized into the experimental group, received an infusion of LOLA, 30 g daily in 500 mL of 5% glucose, for 7 consecutive days. At days 1, 4, and 7, post-TIPS patients in the LOLA group had greater improvement in psychometric tests such as the number connection test (*P* = .001, *P* < .001, *P* < .001), serial dotting test (*P* = .001, *P* = .002, *P* < .001) and line tracing test (*P* = .002, *P* = .002, *P* = .003) compared to patients in control group. Fewer patients in the LOLA group had overt post-TIPS HE compared to control group (n = 1/21 patients with grade 3 HE vs n = 3/19 patients with grade 2 HE, *P* = .331). However, these differences in overt HE were not found to be statistically significant.

### 3.7. Combination therapies: Rifaximin + lactulose

#### 3.7.1. Post-TIPS HE.

Seifert et al (2021) conducted a retrospective review where a combination of lactulose and rifaximin (LR) was administered 72 hours before TIPS and for up to 12 months after TIPS. Lactulose was dosed individually with the aim of 2 to 3 loose stools per day. Rifaximin was administered twice daily at a dosage of 550 mg. Patients in the LR group had less occurrences of post-TIPS HE within 12 months than patients in the no medication group or the lactulose monotherapy group (28.1% vs 52.1%, *P* = .004).

### 3.8. Combination therapies: Rifaximin + lactulose + LOLA

#### 3.8.1. Post-TIPS HE.

Seifert et al (2021) showed that patients receiving a combination of lactulose, rifaximin and LOLA did not have significantly lower occurrences of post-TIPS HE within 12 months compared to patients receiving the combination of lactulose and rifaximin alone (25% vs 29.7%, *P* = .780).

### 3.9. Rifaxmin meta-analysis

#### 3.9.1. Incidence of HE.

**Figure 2.**
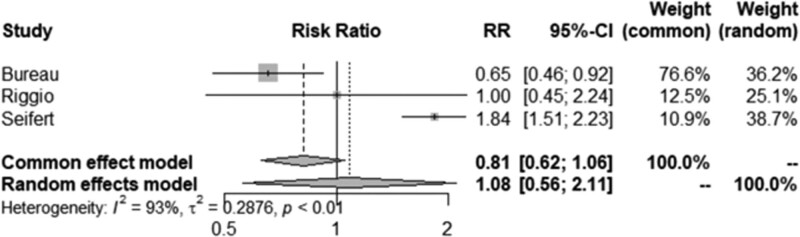
Forest plot of incidence of HE in rifaximin prophylaxis studies. HE = hepatic encephalopathy.

**Figure 3.**
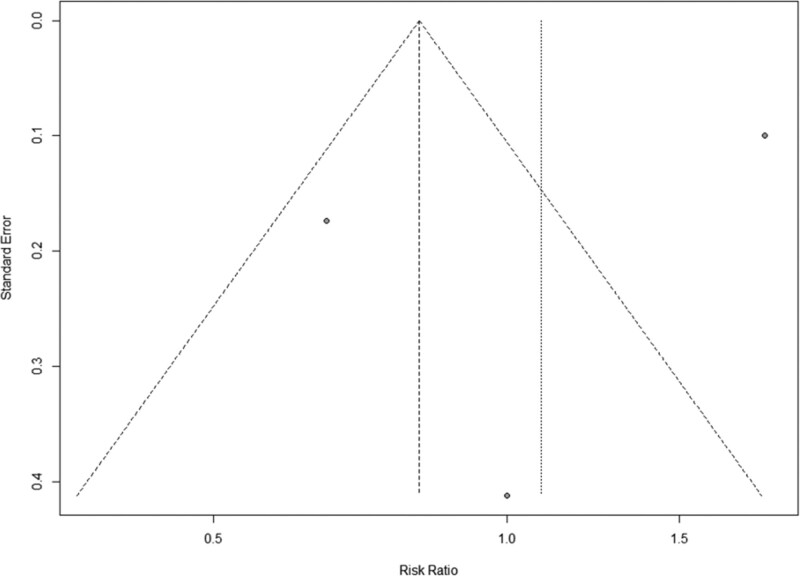
Funnel map of incidence of HE in rifaximin prophylaxis studies. HE = hepatic encephalopathy.

The 3 studies that looked at the number of participants with post-TIPS HE in the control group and rifaximin group allowed for a dichotomous meta-analysis (n = 3) of the effects of rifaximin prophylaxis on incidence of HE. This analysis was possible in 3 studies (Bureau, Riggio, and Seifert) that included 325 patients. The Mantel-Haenszel test found that there was overall no significant difference between the rifaximin-treated group and the control group (RR = 1.08, 95% Cl = 0.56–2.11, *P* = .81) (Fig. 2). A random-effects model was used due to significant heterogeneity between studies (*I*^2^ = 92.7%, *P* < .01) which can arise from differences in settings and participants. The funnel plot also demonstrates potential publication bias or other small study effects (Fig. 3).

## 4. Discussion

Hepatic encephalopathy is a common complication after TIPS placement. Post-TIPS HE can occur in up to 50% of patients and present as covert cognitive impairment to coma. HE is associated with increased hospitalizations and poor prognosis, with up to 40% of patients dying within 12 months of developing HE.^[21]^ Therefore, guidelines should be established to reduce the occurrence of HE in high-risk patients, such as those post-TIPS placement.

Five studies were reviewed in this systematic review. As the included studies investigated a number of different medications that are not comparable to each other, rifaximin was the only medication that was eligible for completion of meta-analysis. There were 3 studies that reported the effect of rifaximin on incidence of HE included in the meta-analysis. The data for the other medications and various combinations were therefore summarized narratively and their qualities were assessed using GRADE. Literature suggests rifaximin alone is not effective for primary prophylaxis of overt HE.^[22]^ Our meta-analysis that included 3 studies by Bureau, Riggio, and Seifert did not find any association of rifaximin prophylaxis with reduction in post-TIPS HE. However, given that only 3 studies are included and that there was considerable heterogeneity of 93%, there is not enough evidence to draw a strong conclusion. We therefore focused on a mainly our narrative review to summarize existing evidence. Two studies included in the review also concluded that rifaximin monotherapy was not effective in reducing post-TIPS HE occurrence,^[11,20]^ whereas 1 study concluded rifaximin prophylaxis was effective in reducing grade 3 and 4 post-TIPS HE, rehospitalization and mortality.^[19]^ However, in this study, patients were started on rifaximin therapy 2 weeks prior to the TIPS procedure, a timeframe which was started earlier than in the patients in the other 2 studies (i.e., 72 hours prior and no prior administration at all). The differences in the timeframe under which rifaximin prophylaxis was started may contribute to the differences in the conclusions made by the 3 studies.

Other than rifaximin monotherapy, the other monotherapies of lactulose, lactitol, LOLA, and albumin, were each reviewed in a single study. These studies results did not find significant difference in the occurrence of post-TIPS HE with prophylactic medication. Outside the context of TIPS, studies have shown lactulose to be an effective prophylactic medication to reduce HE occurrence in cirrhotic patients.^[23]^

A combinational therapy using rifaximin and lactulose may be beneficial for reducing post-TIPS HE as Seifert et al (2021) demonstrated that treating patients with lactulose and rifaximin reduced occurrence of post-TIPS HE. This finding is in-line with prior literature that has shown that the addition to rifaximin to lactulose reduces occurrence of overt HE and HE-related hospitalizations when compared to lactulose alone.^[24]^

To the best of our knowledge, our study is the first systematic review that examines different medical interventions for post-TIPS HE prophylaxis. Although the scarcity of current data meant a meta-analysis could not be completed for most of the medications, we aimed to make our qualitative analysis as objective and comprehensive as possible by adhering to predefined criteria using synthesis without meta-analysis guidelines when providing narrative descriptive synthesis, as well as using GRADE to assess the certainty of evidence. The studies analyzed in our review also had strong internal validity, with our quality assessment demonstrating that 2 RCTs had low risk of bias, and 1 RCT and 2 cohort studies had moderate risk of bias. That being said, the major limitation of this review is the limited number of studies currently available in literature. For all monotherapies other than rifaximin, there was only 1 study that met the inclusion criteria. Furthermore, some results reported were based on small sample sizes and did not report secondary outcomes such as all-cause mortality.

Our study highlights the need to continue research into the area of medical prophylaxis against post-TIPS HE is an understudied topic. Although existing literature regarding medical treatment of HE is abundant, it is evident that their efficacy cannot be assumed when used prophylactically for patients post-TIPS placement specifically. Our review demonstrates that certain medications or their combination therapy, such as rifaximin and lactulose, would be worth investigating further to establish whether they truly have a role as a medical prophylaxis for post-TIPS HE.

As such, we hope that we will be able to make stronger recommendations in the future once the available data becomes more robust. The ongoing The Prevention of hepatic Encephalopathy by Administration of Rifaximin and Lactulose in patients with liver cirrhosis undergoing placement of a TIPS (PEARL) trial will provide valuable data as it is a multi-center RCT that will have a total sample size is 238 patients.^[25]^ As most monotherapies investigated by the studies included in our review did not demonstrate efficacy, it would also be interesting to see if there are other unstudied pharmacotherapy that could play a prophylactic role for post-TIPS HE. One notable ongoing study is the hepatic encephalopathy prevention with polydextrose after TIPS (POEME) pilot study, which will investigate the role of prophylactic treatment with polydextrose, a nondigestible oligosaccharide often used as a prebiotic.^[26]^

## 5. Conclusion

Our systematic review examined 5 studies that reported on the use of various medical pharmacotherapy for prophylaxis of post-TIPS HE. A meta-analysis was done for the 3 studies that examined rifaximin monotherapy. Narrative review of included studies did not find prophylaxis with any monotherapy (rifaximin, lactulose, lactitol, LOLA, or albumin) to be associated with reduction in HE occurrence. Our meta-analysis did not find a significance difference when using rifaximin monotherapy. However, only 3 studies were eligible to be included in the review. Further research would still be worthwhile to increase sample size. As well, a combination of rifaximin and lactulose could potentially be associated with lower HE occurrence based on 1 study by Seifert et al, making it another potential area of investigation. While the limitations in available data hinders any strong recommendations based on the results of this review, our review summarizes the extent of current research on this topic to highlight the current gaps in knowledge and identify medical therapies worth further investigations.

## Author contributions

**Conceptualization:** Aileen Liang, Sukhman Brar, Majed Almaghrabi, Anouar Teriaky.

**Data curation:** Aileen Liang, Sukhman Brar.

**Formal analysis:** Aileen Liang, Sukhman Brar.

**Investigation:** Aileen Liang, Sukhman Brar, Majed Almaghrabi, Anouar Teriaky.

**Methodology:** Aileen Liang, Sukhman Brar, Majed Almaghrabi, Anouar Teriaky.

**Project administration:** Aileen Liang, Sukhman Brar, Majed Almaghrabi, Anouar Teriaky.

**Resources:** Aileen Liang, Sukhman Brar.

**Software:** Aileen Liang, Sukhman Brar.

**Supervision:** Majed Almaghrabi, Anouar Teriaky.

**Validation:** Aileen Liang, Sukhman Brar.

**Visualization:** Aileen Liang, Sukhman Brar.

**Writing – original draft:** Aileen Liang, Sukhman Brar.

**Writing – review & editing:** Aileen Liang, Sukhman Brar, Majed Almaghrabi, Mohammad Qasim Khan, Karim Qumosani, Anouar Teriaky.

## Supplementary Material




