# Supplementary material for: Primary prevention of hepatic encephalopathy post-TIPS: A systematic review and meta-analysis
Source: Medicine (Baltimore). 2023 Sep 22;102(38):e35266. doi: 10.1097/MD.0000000000035266 (PMC10519530; doi:10.1097/MD.0000000000035266)
Supplement: Supplementary file 1 [file medi-102-e35266-s001.docx]

**Appendix 1: Search strategy**

Date cutoff: July 1, 2023

- Concept 1: hepatic encephalopathy
- Concept 2: TIPS
- Concept 3: pharmacological prevention (eg. Lactulose, rifaximin)

| Database | # of results: concepts 1+2+3 |
| --- | --- |
| Medline | 156 |
| EMBASE | 695 |
| Scopus | 564 |
| Cochrane | 54 |
| CINAHL | 22 |

Medline

| Concept 1: Hepatic Encephalopathy | Concept 2: TIPS | Concept 3: pharmacological prevention |
| --- | --- | --- |
| exp Hepatic Encephalopathy/  OR  (hepatic coma* or hepatic encephalopath* or portal-systemic encephalopath* or portalsystemic encephalopath* or hepatic encephalopath* or hepatocerebral encephalopath* or hepatic stupor*).tw,kf. | exp Portasystemic Shunt, Transjugular Intrahepatic/  OR  (transjugular intrahepatic portosystemic shunt or portosystemic shunt or tips procedure or tipss procedure).tw,kf. | exp Pharmaceutical Preparations/  OR  exp Lactulose/  OR  exp Rifaximin/  OR  (pharmacotherap* or drug or rifaximin or lactulose or nonabsorbable disaccharide* or probiotic* or glutaminase inhibitor* or neomycin or metronidazole or albumin or branched-chain amino acid* or metabolic ammonia scavenger* or ornithine phenylacetate or glyceryl phenylbutyrate or l-ornithine l-aspartate).tw,kf. |

EMBASE

| Concept 1: Hepatic Encephalopathy | Concept 2: TIPS | Concept 3: pharmacological prevention |
| --- | --- | --- |
| exp hepatic nncephalopathy/  OR  (hepatic coma* or hepatic encephalopath* or portal-systemic encephalopath* or portalsystemic encephalopath* or hepatic encephalopath* or hepatocerebral encephalopath* or hepatic stupor*).tw,kw. | exp Transjugular Intrahepatic portosystemic shunt/  OR  (transjugular intrahepatic portosystemic shunt or portosystemic shunt or tips procedure or tipss procedure).tw,kw. | exp drug therapy/  OR  exp lactulose/  OR  exp rifaximin/  OR  (pharmacotherap* or drug or rifaximin or lactulose or nonabsorbable disaccharide* or probiotic* or glutaminase inhibitor* or neomycin or metronidazole or albumin or branched-chain amino acid* or metabolic ammonia scavenger* or ornithine phenylacetate or glyceryl phenylbutyrate or l-ornithine l-aspartate).tw,kw. |

Scopus

## ( TITLE-ABS-KEY ( "hepatic coma*" OR "hepatic encephalopath*" OR "portal-systemic encephalopath*" OR "portalsystemic encephalopath*" OR "hepatic encephalopath*" OR "hepatocerebral encephalopath*" OR "hepatic stupor*" ) AND TITLE-ABS-KEY ( "transjugular intrahepatic portosystemic shunt" OR "portosystemic shunt" OR "tips procedure" OR "tipss procedure" ) AND TITLE-ABS-KEY ( "pharmacotherap*" OR "drug" OR "rifaximin" OR "lactulose or nonabsorbable disaccharide*" OR "probiotic*" OR "glutaminase inhibitor*" OR "neomycin" OR "metronidazole" OR "albumin" OR "branched-chain amino acid*" OR "metabolic ammonia scavenger*" OR "ornithine phenylacetate" OR "glyceryl phenylbutyrate" OR "l-ornithine l-aspartate" ) )

Cochrane

| Concept 1: Hepatic Encephalopathy | Concept 2: TIPS | Concept 3: pharmacological prevention |
| --- | --- | --- |
| (“hepatic encephalopathy” OR “hepatic coma” OR “portal-systemic encephalopathy” OR “hepatocerebral encephalopathy” OR “hepatic stupor”) | ("transjugular intrahepatic portosystemic shunt" OR "portosystemic shunt" OR tips) | (Lactulose or "Nonabsorbable Disaccharide*" OR Rifaximin OR Antibiotic OR Probiotic OR Neomycin OR Metronidazole OR Albumin OR “glutaminase inhibitor” OR "Ornithine phenylacetate" OR "Glyceryl phenylbutyrate" OR "Metabolic Ammonia Scavenger" OR "L-ornithine L-aspartate" OR Flumazenil OR "branched-chain amino acid*" OR Pharmaceutical) |

CINHL

| Concept 1: Hepatic Encephalopathy | Concept 2: TIPS | Concept 3: pharmacological prevention |
| --- | --- | --- |
| (MH "Hepatic Encephalopathy") OR "(“hepatic encephalopathy” OR “hepatic coma” OR “portal-systemic encephalopathy” OR “hepatocerebral encephalopathy” OR “hepatic stupor”)" | (MH "Portasystemic Shunt, Surgical") OR "transjugular intrahepatic portosystemic shunt" | (MH "Rifaximin") OR "rifaximin"  OR "Lactulose" or "Nonabsorbable Disaccharide"  OR  (MH "Probiotics") OR "Probiotic"  OR  (MH "Neomycin") OR "Neomycin"  OR  (MH "Metronidazole") OR "Metronidazole"  OR  (MH "Serum Albumin") OR (MH "Albumins") OR "albumin"  OR  "glutaminase inhibitors"  OR  "Ornithine phenylacetate"  OR  "Glyceryl phenylbutyrate"  OR "Glyceryl phenylbutyrate"  OR  "Metabolic Ammonia Scavenger"  OR  "L-ornithine L-aspartate"  OR  (MH "Flumazenil") OR "Flumazenil"  OR  (MH "Amino Acids, Branched-Chain") OR "branched-chain amino acid"  OR  (MH "Drugs") OR "drugs"  OR  (MH "Drug Therapy") OR "pharmacotherapy" |
