# Supplementary figures and images for: Primary prevention of hepatic encephalopathy post-TIPS: A systematic review and meta-analysis
Source: Medicine (Baltimore). 2023 Sep 22;102(38):e35266. doi: 10.1097/MD.0000000000035266 (PMC10519530; doi:10.1097/MD.0000000000035266)

**Appendix 2: Quality Assessment Plots**

RoB-2


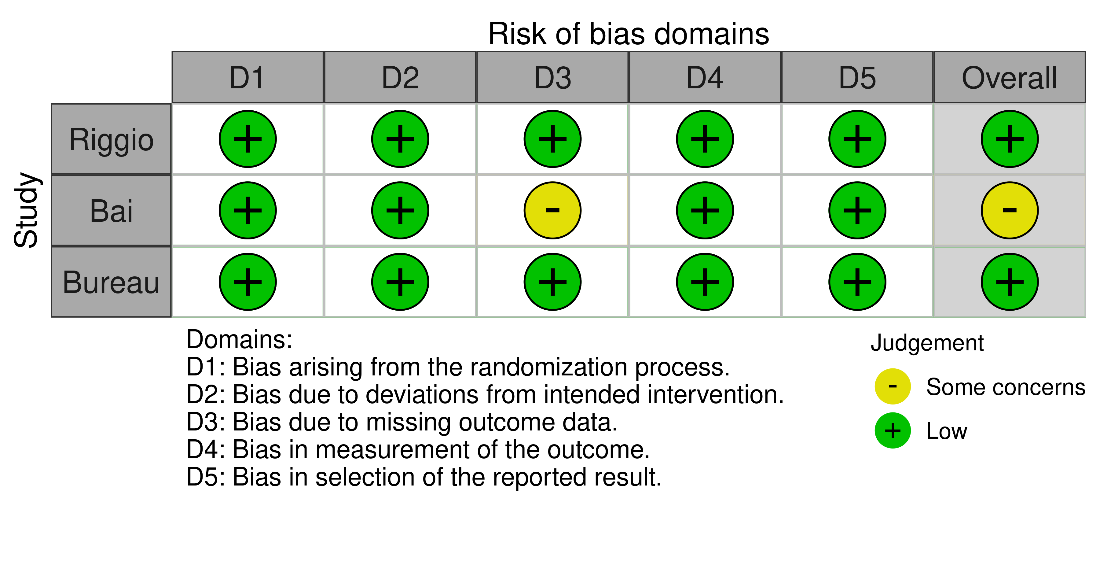


ROBINS-I


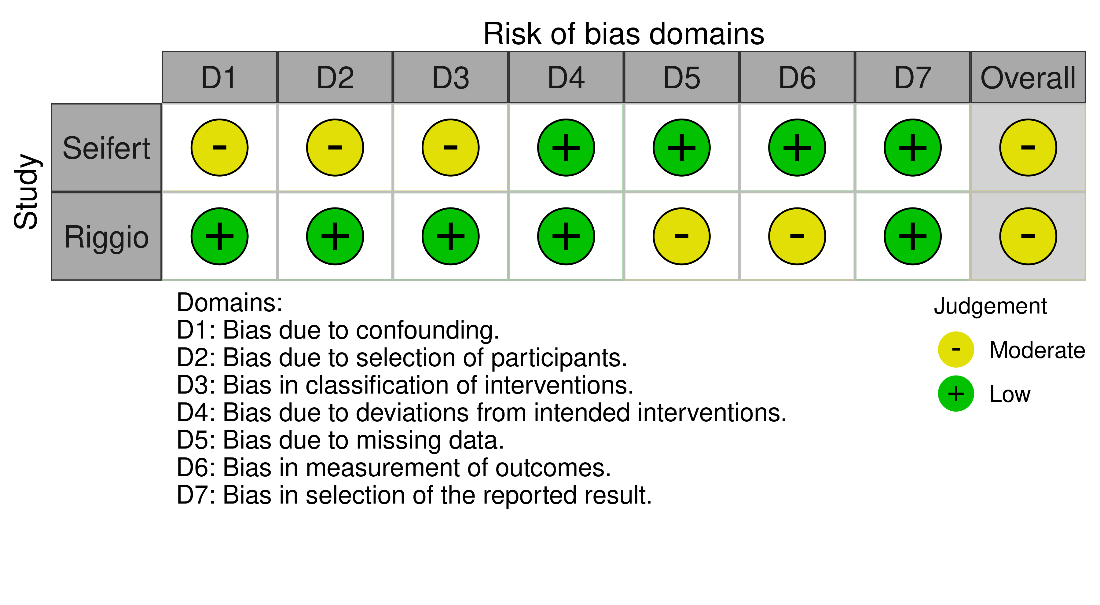

Supplement: Supplementary file 2 [file medi-102-e35266-s002.docx]
